# Prevalence of Bacterial Vaginosis and Associated Risk Factors among Women Complaining of Genital Tract Infection

**DOI:** 10.1155/2017/4919404

**Published:** 2017-08-02

**Authors:** Adane Bitew, Yeshiwork Abebaw, Delayehu Bekele, Amete Mihret

**Affiliations:** ^1^Department of Medical Laboratory Sciences, College of Health Sciences, Addis Ababa University, P.O. Box 1176, Addis Ababa, Ethiopia; ^2^Clinical Laboratory, Fitche Hospital, P.O. Box 46, Oromia Administrative Region, Ethiopia; ^3^Department of Obstetrics and Gynecology, St Paul's Hospital Millennium Medical College, P.O. Box 1271, Addis Ababa, Ethiopia; ^4^Department of Clinical Bacteriology and Mycology, Ethiopian Public Health Institute, P.O. Box 1242, Addis Ababa, Ethiopia

## Abstract

**Background:**

Bacterial vaginosis is a global concern due to the increased risk of acquisition of sexually transmitted infections.

**Objectives:**

To determine the prevalence of bacterial vaginosis and bacteria causing aerobic vaginitis.

**Methods:**

A cross-sectional study was conducted among 210 patients between September 2015 and July 2016 at St. Paul's Hospital. Gram-stained vaginal swabs were examined microscopically and graded as per Nugent's procedure. Bacteria causing aerobic vaginitis were characterized, and their antimicrobial susceptibility pattern was determined.

**Results:**

The overall prevalence of bacterial vaginosis was 48.6%. Bacterial vaginosis was significantly associated with number of pants used per day (*p* = 0.001) and frequency of vaginal bathing (*p* = 0.045). Of 151 bacterial isolates, 69.5% were Gram-negative and 30.5% were Gram-positive bacteria. The overall drug resistance level of Gram-positive bacteria was high against penicillin, tetracycline, and erythromycin. Cefoxitin and tobramycin were the most active drugs against Gram-positive bacteria. The overall drug resistance level of Gram-negative bacteria was high against tetracycline, ampicillin, and amoxicillin. Amikacin and tobramycin were the most active drugs against Gram-negative bacteria.

**Conclusions:**

The prevalence of bacterial vaginosis was high and was affected by individual hygiene. Routine culture of vaginal samples should be performed on patients with vaginitis and the drug susceptibility pattern of each isolate should be determined.

## 1. Introduction

Vaginitis is an inflammation of the vagina in which bacterial vaginosis, vulvovaginal candidiasis, and trichomoniasis are the most vaginitides [1]. Nearly 5–10 million females seek gynecologic advice for vaginitis every year worldwide [2].

Bacterial vaginosis is described as a shift in the balance of the vaginal microflora characterized by an increase in the vaginal pH, a reduction in lactobacilli, predominantly hydrogen peroxide producing species, and an increase in facultative and anaerobic bacteria in number and/or type [3]. Although the prevalence of bacterial vaginosis differs widely from country to country within the same region and even within similar population groups, it has been estimated to be in the range of 8% to 75% [4]. Bacterial vaginosis can occur in any age group, but globally it is more prevalent in females of reproductive age [1].

For many years, bacterial vaginosis has received little attention, since it is considered to be a trivial disease. However, it is a morbid disease in terms of loss of working days and treatment cost [2]. Furthermore, it increases the risk of acquiring (i) human immunodeficiency virus [5] and other sexually transmitted infections (STI), such as gonorrhea, trichomoniasis, and herpes simplex virus type 2 (HSV-2) [6, 7], and (ii) miscarriage, preterm labor, preterm delivery, and postpartum complications such as endometritis and wound infections in pregnant women [8–11]. It also increases HIV viral shedding [12, 13].

The diagnosis of bacterial vaginosis can be made using clinical criteria [14] or in the laboratory by scoring bacterial morphotypes from a Gram stain of vaginal fluid, where a score of 0–3 represented normal vaginal flora, a score of 4–6 represented intermediate vaginal flora, and a score of 7–10 was considered as diagnostic for bacterial vaginosis [15]. A few studies [16, 17] have also isolated and characterized aerobic microorganisms identified as major causes of aerobic vaginitis from cultures of vaginal swabs.* E. coli, Pseudomonas *spp.*, S. aureus, Mycoplasma hominis, and Ureaplasma urealyticum *have been reported as the most frequently isolated microorganisms from patients with aerobic vaginitis.

Although bacterial vaginosis is associated with numerous health problems and is a major global concern, it has been the focus of neither intensive study nor active control programs in Ethiopia. Therefore, the purpose of the present study was to determine the prevalence of bacterial vaginosis and associated risk factors among women attending gynecology and antenatal clinics.

## 2. Materials and Methods

### 2.1. Study Design, Period, and Area

A hospital-based cross-sectional study was conducted from September 2015 to July 2016 at Department of Obstetrics and Gynecology, St Paul's Hospital Millennium Medical College, Addis Ababa, Ethiopia. The hospital has 1486 professional and supporting staff members. It provides health services for about 700 patients daily. It has 13 departments and 340 beds offering various specialized services. The Gynecology Department has 5 OPD clinics; and many patients are referred from all over the country to this hospital.

### 2.2. Inclusion and Exclusion Criteria

All women willing to participate in the study with presumptive diagnosis of vaginal infections and no history of antibacterial therapy within two weeks prior to their attendance constituted the inclusion criteria. Women with genital malignancy were excluded from the study. The requisition form filled out by physicians was used as standard proforma to document clinical information and previous treatment history.

### 2.3. Collection of Sociodemographic Characteristics, Sexual Behavior, and Reproductive Health Information

Sociodemographic characteristics, sexual behavior, and reproductive health characteristics such as age, educational and marital status, number of lifetime male sex partners, history of abortion, previous history of genital tract infection, frequency of vaginal bathing, and pant change were collected by face-to-face interviews using a structured questionnaire.

### 2.4. Specimen Collection and Transportation

Upon admission to the study, physicians performed clinical examination of each participant and recorded signs of vaginal abnormalities. During examinations, vaginal specimens were collected aseptically from the study participants using sterile rayon-tipped applicator stick swabs by experienced nurses. All vaginal swabs were then transferred without delay to the microbiology laboratory of the Ethiopian Public Health Institute.

### 2.5. Microscopic Examination

For diagnosis of bacterial vaginosis, slide smears were prepared from vaginal swabs, and the slides were heat-fixed, Gram-stained, and examined under oil immersion objective. Each slide was then graded as per the standardized quantitative morphological classification method developed by Nugent et al. [15] which assigns a score between 0 and 10 based on the following various bacterial morphotypes: large Gram-positive rods (*Lactobacillus* morphotypes), small Gram-variable rods (*G. vaginalis* morphotypes), small Gram-negative rods (*Bacteroides *spp. morphotypes), curved Gram-variable rods (*Mobiluncus *spp. morphotypes), and Gram-positive cocci. Each morphotype was quantitated from 1 to 4+ with regard to the number of morphotypes per oil immersion field (0, no morphotypes; 1+, less than 1 morphotype; 2+, 1 to 4 morphotypes; 3+, 5 to 30 morphotypes; and 4+, 30 or more morphotypes). Scores between 0 and 3 represented “normal vaginal flora,” scores between 4 and 6 represented “intermediate vaginal flora,” and scores between 7 and 10 were considered diagnostic for bacterial vaginosis.

### 2.6. Inoculation and Incubation

Each vaginal swab was inoculated onto Blood Agar base (Oxoid, Basingstoke, Hampshire, UK) to which 10% sheep blood was incorporated, MacConkey agar (Oxoid, Basingstoke, Hampshire, UK), and chocolate agar (Oxoid, Basingstoke, Hampshire, UK) before slide preparation for the isolation and characterization of aerobic bacteria. Blood agar and chocolate agar plates were incubated at 35–37°C up to 48 hrs in a 5% CO_2_ incubator. MacConkey agar was incubated at 35–37°C up to 48 hrs aerobically. Preparation and performance evaluation of culture media were done as per the instruction of the manufacturer.

### 2.7. Bacterial Identification

Pure isolates of bacterial pathogen were preliminarily characterized by colony morphology, Gram stain, and hemolytic reactions on blood agar plates. Identification of bacteria to genus and/or species level was done by employing an array of routine biochemical tests such as DNase, catalase, optochin, bacitracin, CAMP, and bile-esculin tests for Gram-positive bacteria and indole production, H_2_S production, gas production, motility, urease, citrate utilization tests, and fermentation of various carbohydrates for Gram-negative bacteria.

### 2.8. Antimicrobial Susceptibility Testing

The in vitro antibacterial susceptibility testing of bacterial isolates was performed by the Kirby-Bauer disc diffusion method. The following antimicrobial agents were employed: penicillin (10 *μ*g), cefoxitin (30 *μ*g), trimethoprim/sulfamethoxazole (25/23.75 *μ*g), ceftriaxone (30 *μ*g), clindamycin (2 *μ*g), erythromycin (15 *μ*g), gentamycin (10 *μ*g), ciprofloxacin (5 *μ*g), tobramycin (10 *μ*g), vancomycin (10 *μ*g), tetracycline (30 *μ*g), amoxicillin (10 *μ*g), amoxicillin/clavulanate (20/10 *μ*g), and amikacin (30 *μ*g). Sensitivity test results were interpreted according to the Clinical and Laboratory Standards Institute. Reference strains,* E. coli* ATCC 25922 and* S. aureus* ATCC 25923, were used for quality control for antimicrobial susceptibility testing. All drugs were generously provided by the Ethiopian Public Health Institute.

### 2.9. Statistical Analysis

All data from the investigation were coded, double-entered, and analyzed using SPSS version 20. Descriptive statistics and logistical regressions were used to estimate crude and adjusted crude ratio with 95% confidence interval to the different variables. *p* value < 0.05 was considered significant.

### 2.10. Ethical Clearance

All ethical considerations and obligations were duly addressed, and the study was conducted after the approval of the Department Research and Ethical Review Committee (DRERC) of the Department of Medical Laboratory Sciences, College of Health Sciences, Addis Ababa University, and Ethical Review Board of the hospital. Written informed consent was obtained from the participants before data collection. Each respondent was given the right to refuse to take part in the study and to withdraw at any time during the study period. All information obtained from the study subjects was coded to maintain confidentially. When the participants were found to be positive for a bacterial pathogen, they were informed by the hospital clinician and received proper treatment. An assent form was completed and signed by a family member and/or adult guardian for participants under the age of 16 years.

## 3. Results

### 3.1. Prevalence of Bacterial Vaginosis with Sociodemographic Characteristics, Sexual Behavior, Reproductive Health Information, and Personal Hygiene

A total of 210 women were included in the study. The overall prevalence of bacterial vaginosis was 48.6%. Subgroup prevalence of bacterial vaginosis is presented in Table 1. Younger women, 15 to 24 years old, had somewhat lower prevalence (41.5%) of bacterial vaginosis, while in the 25 years and older group, the prevalence was between 47.8% and 60.0%. As shown in Table 1, the adjusted odds ratio depicted that bacterial vaginosis was not significantly associated with age.

The prevalence of bacterial vaginosis varied with education and marital status. Women with a college-level education were less likely to be positive for bacterial vaginosis than those with a high school education or less (35.9% versus 44.7–55.3%). Similarly, the prevalence of bacterial vaginosis was high among unmarried study subjects (53.8%) compared to those who were married (44.8%) or divorced (50.0%). As depicted in Table 1, neither marital status nor education was statistically associated with bacterial vaginosis.

The prevalence of bacterial vaginosis varied with selected reproductive health history. For example, it was less prevalent in patients who had previous bacterial vaginosis (46.3%) than in patients with no previous bacterial vaginosis (50.4%). The prevalence rate of bacterial vaginosis was higher in women with a history of abortion (53.8%) than in women with no history of abortion (46.8%). These two variables were not significantly associated with bacterial vaginosis (Table 1). The prevalence of bacterial vaginosis also varied with number of lifetime male sex partners. Women who reported 1–3 lifetime male sex partners had prevalence rate of 43.4%, while those who reported ≥4 lifetime male sex partners had prevalence rate of 58%. The prevalence of bacterial vaginosis was lower among patients who changed pants more frequently (two per day; 36.9%) than among those who changed their pants less frequently (one pant for 2–4 days; 57.6%). Statistical analysis showed a significant correlation between bacterial vaginosis and number of pants used per day (*p* = 0.001). Similarly, patients who bathed their vaginal region more frequently were less affected than those who did not bath their vaginal area as much (prevalence rate of 40.2% versus 53.9%). The association of frequency of vaginal bathing and bacterial vaginosis was statistically significant (*p* = 0.045).

### 3.2. Spectrum of Bacteria Causing Aerobic Vaginosis

A total of 151 bacterial isolates were recovered from vaginal swabs, of which 105 (69.5%) were Gram-negative and 46 (30.5%) were Gram-positive bacteria. Of the Gram-negative bacteria,* E. coli *and* Klebsiella *spp. were dominant.* S. aureus* and* S. agalactiae *were the dominant Gram-positive bacteria (Table 2).

### 3.3. Drug Susceptibility Pattern of Bacterial Isolates

Table 3 summarizes the overall drug susceptibility pattern of the Gram-positive bacteria against the eleven antibacterial drugs tested. Among the agents tested, the highest overall resistance rate of Gram-positive bacteria was observed against penicillin (67.4%), followed by tetracycline (58.7%) and erythromycin (45.6%). Cefoxitin and tobramycin were the most active of the drugs tested against Gram-positive bacteria.* S. aureus,* the most frequently isolated Gram-positive bacterium, was 97.2%, 88.8%, and 86.1% sensitive to cefoxitin, tobramycin, and clindamycin, respectively.

The overall drug susceptibility pattern of Gram-negative bacteria against the nine antibacterial agents tested is summarized in Table 4. Tetracycline exhibited the highest overall drug resistance rate (77.3%) against Gram-negative bacteria, followed by ampicillin (77.1) and amoxicillin (70.6%). Amikacin with an overall sensitivity rate of 85.7% and tobramycin with an overall sensitivity rate of 82.8% were better active against Gram-negative bacteria. As far as species-specific antimicrobial resistance rates are concerned,* E. coli*, the most frequently isolated bacterium, showed 76.7% resistance to both ampicillin and tetracycline. The lowest resistance rate was observed with amikacin and tobramycin. Amikacin, tobramycin, and gentamycin were the most active drugs against* K. pneumoniae,* the second most commonly isolated Gram-negative bacterium.

## 4. Discussion

The overall prevalence rate of bacterial vaginosis in the present study as determined by Gram-stain Nugent scoring criteria was 48.6%. Although the prevalence rate of bacterial vaginosis in the present study was well within the reported range, that is, 8%–75% [4], it was higher than the prevalence rates of bacterial vaginosis reported by similar local studies [16, 18]. Local studies reported prevalence rates of bacterial vaginosis in the range of 15.4% [16] to 19.4% [18]. Lower prevalence rates of bacterial vaginosis than those in the present study were also reported from other sub-Saharan countries, such as Kenya (37%), Botswana (38%), and Zimbabwe (32.5%) [19–21]. Sociodemographic characteristics, sexual activity, reproductive health information, and behavioral and genital hygiene have been identified as causes of variation in the prevalence rates of bacterial vaginosis [22–26].

The present study revealed that the proportion of bacterial vaginosis was the highest in age groups above 45 years. Our finding was comparable to the studies of Fang et al. [27], Ocviyanti et al. [28], and Yusuf et al. [29]. Similarly, a study conducted in a population of individuals seeking STD treatment showed that 23% of women aged 14–24 years exhibited bacterial vaginosis compared to 33% of women aged 25 years and older [30]. An elevation of pH in women above age of 45 years has been identified as a cause of a decline in the level of estrogen, which in turn creates an optimal condition for the growth of bacteria other than lactobacilli.

Lack of education has been found to be significantly associated with bacterial vaginosis [26]. However, our finding like other studies [27] contradicted this conclusion. In the present study, bacterial vaginosis was higher among subjects having an education level of primary and secondary school compared to illiterate patients.

The role of sexual activity in the acquisition of bacterial vaginosis is not clear. Bacterial vaginosis' prevalence rates of 18.8%, 18%, and 12% were reported among women who reported that they have never had sex by Koumans et al. [25], Yen et al. [24], and Bump and Buesching [23], respectively. Contrary to this, sexual behavior-related characteristics, including number of lifetime male sex partners, multiple male sex partners, and a recent history of new sex partners, have been consistently associated with bacterial vaginosis. In support of a role of sexual transmission, Bump and Buesching [23], Yen et al. [24], and Allsworth and Peipert [31] found that multiple or new sex partners increased the risk of acquiring bacterial vaginosis by a factor of 1.6–2.5. These studies also suggested that condom use may be protective. In the present study, the adjusted odds ratio analysis revealed that bacterial vaginosis and the number of lifetime male sex partners were not statistically associated (*p* = 0.103). Thus, our result did not support previous studies that reported that multiple or new sex partners increased the risk of acquiring bacterial vaginosis [2, 23, 24, 31]. Verstraelen et al. [32] argue that bacterial vaginosis may be considered as a sexually enhanced disease rather than sexual transmitted infection, with the frequency of sexual intercourse being a critical factor.

In contrast to other findings, no significant correlation was observed between bacterial vaginosis and number of abortions [24]. As far as personal hygiene is concerned, statistical analysis showed a significant correlation between frequency of vaginal bathing (*p* = 0.047) and number of pants used per day (*p* = 0.001). Our result was in good agreement with the findings of Bahram et al. [26] who reported that bacterial vaginosis is significantly associated with individual hygiene.

Bacterial vaginosis is a situation that occurs when lactobacilli are replaced by the overgrowth of anaerobic bacteria, primarily* G. vaginalis* and* Mobiluncus *spp. Other bacteria (*Escherichia coli, Klebsiella* spp.,* Acinetobacter *spp.,* Staphylococcus *spp., enterococci, and* Streptococcus agalactiae* (group B streptococci), however, have been termed “intermediate flora” in some studies or have been included with bacterial vaginosis in others [8, 33]. Still others consider them as distinct bacterial floras that cause aerobic vaginitis which has been thought to be a better candidate than bacterial vaginosis as a cause of pregnancy complications such as preterm rupture of the membranes and preterm delivery [2, 34, 35]. With attention to the above findings, in the present study, vaginal swabs were cultured and 151 bacterial isolates of Gram-positive and Gram-negative bacteria were recovered. The type of bacterial isolates and their frequency recorded in the present study were more diverse than in the study of Lakshmi et al. [1, 16]. Therefore, further studies to differentiate the effects of bacterial vaginosis and aerobic vaginitis on the outcome of pregnancy should be conducted.

The overall drug resistance rates of Gram-negative bacterial isolates ranged from 14.3% for amikacin to 77.3% for tetracycline.* E. coli,* the most frequently isolated Gram-negative bacterium, showed a high level of resistance to tetracycline and ampicillin. Contrary to this, 86% of* E. coli* were susceptible to amikacin and tobramycin. The drug resistance of level of* K. pneumoniae,* the second frequent isolate, was high against ampicillin, amoxicillin, and tetracycline.

Similarly, the overall drug resistance rates of Gram-positive bacterial isolates ranged from 2.8% for cefoxitin to 67.4% for penicillin.* S. aureus, *the most frequently isolated Gram-positive bacterium, revealed a high level of resistance to the commonly prescribed drugs, penicillin, tetracycline, and erythromycin. Our result was consistent with studies conducted in Ethiopia [16, 36, 37] and Pakistan [38, 39]. Availability of antimicrobials without prescription and inappropriate dosing schedules may explain the isolation of high level of drug resistance in the present study.

## 5. Conclusions

The prevalence of bacterial vaginosis was relatively high and was affected by individual hygiene. Therefore, comprehensive healthcare education aimed at reducing bacterial vaginosis is needed. Isolation and characterization of aerobic bacteria implicated in causing aerobic vaginitis initiate inclusion of vaginal culture and sensitivity testing along with microscopic and clinical diagnosis of bacterial vaginitis.

## Figures and Tables

**Table 1:** Prevalence of bacterial vaginosis (Nugent's Gram Stain Score: 7–10) by selected characteristics in relation to sociodemographic characteristics, sexual behavior, reproductive health information, and personal hygiene (*n* = 210).

Characteristics	Number	Bacterial vaginosis (*n*, %)		*p* value	COR	95% CL	*p* value	AOR	95% CL
		Yes	No						
Age in years									
15–24	53 (25.2)	22 (41.5)	31 (58.4)		1			1	
25–44	117 (55.7)	56 (47.8)	61 (52.1)	0.416	.763	0.178–0.955	0.379	0.735	0.371–1.459
45–64	40 (19.04)	24 (60)	16 (40)	0.039	.413	0.397–1.466	0.078	0.457	0.191–1.093
Total	210	102 (48.6)	108 (51.4)						
Marital status									
Unmarried	65 (30.9)	35 (53.8)	30 (46.1)		1				
Married	107 (50.9)	48 (44.8)	59 (55.1)	0.254	1.434	0.772–2.663			
Divorced	38 (18.1)	19 (50)	19 (50)	0.706	1.167	0.524–2.600			
Total	210 (100)	102 (48.6)	108 (51.4)						
Education									
Illiterate	47 (22.4)	21 (44.7)	26 (55.3)	0.410	0.693	0.290–1.657			
Primary school	59 (28.1)	31 (52.5)	28 (47.5)	0.108	0.506	0.221–1.160			
Secondary school	65 (31.0)	36 (55.3)	29 (44.6)	0.056	0.451	0.199–1.021			
College	39 (18.6)	14 (35.9)	25 (64.1)		1				
Total	210 (100)	102 (48.6)	108 (51.4)						
Number of lifetime male sex partners									
1–3	136 (64.8)	59 (43.4)	77 (56.6)		1			1	
≥4	74	43 (58)	31 (41.8)	0.042	1.810	1.021–3.210	0.103	1.645	.904–2.995
Total	210	102 (48.6)	108 (51.4)						
History of abortion									
Yes	52 (24.7)	28 (53.8)	24 (46.1)	0.381	0.755	0.403–1.416			
No	158 (75.2)	74 (46.8)	84 (53.1)						
Previous BV/GTI					1				
Yes	95 (45.2)	44 (46.3)	51 (53.6)	0.552	1.179	0.684–2.033			
No	115 (54.7)	58 (50.4)	57 (49.5)		1				
Total	210	102 (48.6)	108 (51.4)						
Vaginal bathing/day									
1–3	128 (60.9)	69 (53.9)	59 (46)		1			1	
≥4	82 (39)	33 (40.2)	49 (59.7)	0.054	1.737	0.990–3.045	0.045	1.847	1.013–3.370
Total	120	102	108						
Number of pants used/day									
1-2 pants/a day	92 (43.8)	34 (36.9)	58 (63)		1				
1 pant for 2–4 days	118 (56.1)	68 (57.6)	50 (42.3)	0.003	0.431	0.236–0.754	0.001	0.367	0.201–0.672

*Total*	*102*	*108*							

**Table 2:** Distribution of bacterial isolates (*n* = 151).

Species	Number of isolates	% of the total isolates
*S. aureus *	36	23.8
*S. agalactiae*	7	4.6
*S. pyogenes*	3	2.0
*E. coli*	43	28.5
*Klebsiella pneumoniae*	28	18.5
*Klebsiella ozaenae*	4	2.6
*Enterobacter aerogenes *	17	11.3
*Citrobacter freundii*	6	4.0
*Citrobacter diversus*	2	1.3
*Proteus mirabilis*	1	0.7
*Providencia rettgeri*	4	2.6

Total	151	100%

**Table 3:** Percentage of in vitro antibacterial susceptibility pattern of Gram-positive bacteria isolates (*n* = 46).

Species	P	Antibacterial drugs										
		Pen	FOX	E	DA	SXT	TE	CN	VA	CRO	CIP	TOB
*S. aureus *(36)	S	13.9	97.2	41.7	86.1	58.3	11.1	80.6	—	—	77.7	88.8
	I	—	0	8.3	2.8	22.2	22.2	8.3	—	—	0	0
	R	86.1	2.8	50%	11.1	19.4	63.8	11.1	—	—	22.2	12
*S. agalactiae* (7)	S	100	100	85.7	100	57.1	42.8	ND	100	100	—	—
	I	—	0	0	0	0	28.0	—	0	0	—	—
	R	0	0	14.2	0	42.9	28.0	—	0	0	—	—
*S. pyogenes *(3)	S	100	100	66.67	66.67	33.3	33.3	—	100	100	—	—
	I	—	0	0	0	0	0	—	0	0	—	—
	R	0	0	33.3	33.3	66.67	66.67	—	0	0	—	—

Total isolates (46)	S	32.6	97.2	50.0	86.9	56.5	17.3	80.6	100	100	77.7	88.8
	I	—	0	6.0	2.0	17.3	21.7	8.3	0	0	0	0
	R	67.4	2.8	45.6	10.8	26	58.7	11.1	0	0	22.2	12.0

Pen: penicillin; Fox: cefoxitin; SXT: trimethoprim/sulfamethoxazole; CRO: ceftriaxone; CIP: ciprofloxacin; Da: clindamycin; E: erythromycin; CN: gentamicin; TE: tetracycline; TOB: tobramycin; VA: vancomycin; S: sensitive; R: resistance; P: pattern; —: not tested.

**Table 4:** Percentage of in vitro antibacterial susceptibility pattern of Gram-negative bacteria isolates (*n* = 105).

Species	Pattern	Antibacterial drugs								
		AM	AMC	SXT	TE	CN	CRO	CIP	TOB	AK
*E. coli* (43)	S	23.3	32.6	60.5	18.6	83.7	76.7	79.1	86.04	86.04
	I	0	7	16.3	4.7	0	0	7.0	0	0
	R	76.7	60.5	23.3	76.7	16.3	23.3	14.0	13.96	13.96
*K. pneumoniae *(28)	S	14.3	14.3	14.3	17.8	78.5	60.7	57.1	78.5	82.1
	I	7.1	7.1	0	0	0	0	0	0	0
	R	78.6	78.6	85.7	82.1	21.1	39.0	42.8	21.1	17.8
*K. ozaenae *(4)	S	0	0	0	0	50.0	50.0	50.0	75.0	100
	I	0	0	0	0	50.0	0	0	0	0
	R	100	100	100	100	0	50.0	50.0	25.0	0
*E. aerogenes *(17)	S	0	17.6	28.1	5.9	70.6	70.6	58.8	76.7	82.35
	I	17.6	11.8	18.8	17.6	5.9	11.8	0	0	0
	R	82.4	70.6	53.1	76.5	23.5	17.6	41.2	23.5	17.64
*C. freundii *(6)	S	0	0	50.0	33.33	83.3	66.6	83.3	100	100
	I	0	0	0	0	0	0	0	0	0
	R	100	100	50.0	66.6	16.67	33.33	16.67	0	0
*C. diversus *(2)	S	0	0	0	0	50.	50.0	50.0	50.0	50.0
	I	0	0	0	0	0	0	0	0	0
	R	100	100	100	100	50.0	50.0	50.0	50.0	50.0
*P. mirabilis *(1)	S	100	100	100	100	100	100	100	100	100
	I	0	0	0	0	0	0	0	0	0
	R	0	0	0	0	0	0	0	0	0
*P. rettgeri *(4)	S	100	50.0	50.0	50.0	100	75.0	100	100	100
	I	0	0	0	0	0	0	0	0	0
	R	0	50.0	50.0	50.0	0	25.0	0	0	0

Total isolates	S	18	22.8	39	18	79	68.5	69.5	82.8	85.7
	I	4.7	6.6	9.5	4.7	2.8	1.9	2.8	0	0
	R	77.1	70.6	51.5	77.3	18.2	29.6	27.7	17.2	14.3

AMP: ampicillin; AMC: amoxicillin/clavulanate; SXT: trimethoprim/sulfamethoxazole; CRO: ceftriaxone; CIP: ciprofloxacin; CN: gentamicin; TE: tetracycline; TOB: tobramycin; AK: amikacin; S: sensitive; R: resistance; P: pattern.
